# The Activation of PDGFRβ on Mononuclear Stromal/Tumor Cells in Giant Cell Tumor of Bone After Denosumab Treatment. An Immunohistochemical Study of Five Cases

**DOI:** 10.3389/pore.2022.1610633

**Published:** 2022-08-24

**Authors:** Imre Antal, Zsuzsanna Pápai, Miklós Szendrői, Tamás Perlaky, Katalin Dezső, Zoltán Lippai, Zoltán Sápi

**Affiliations:** ^1^ Department of Orthopaedics, Semmelweis University, Budapest, Hungary; ^2^ Department of Oncology, Hungarian Defence Forces Medical Center, Budapest, Hungary; ^3^ Department of Pathology and Experimental Cancer Research, Semmelweis University, Budapest, Hungary

**Keywords:** giant cell tumor of bone, denosumab, PDGFRβ, combination therapy, sunitinib

## Abstract

Due to the relatively high recurrence rate and the destructive nature of the tumor, the treatment of giant cell tumor is still a challenge. Denosumab appeared to be a promising candidate as a therapeutic drug. However, several studies have reported that tumors can recur during/after treatment with denosumab. Based on activated receptor tyrosine kinase signaling pattern of the stromal/tumor cells, a combination treatment with denosumab and sunitinib has recently been proposed to inhibit recurrences. This prompted us to investigate the PDGFRβ expression of five denosumab treated cases using both primary and recurrent tumors during and after denosumab treatment. In addition, to recognise morphological changes, immunohistochemical analysis of H3F3A and PDGFRβ was also performed. As an effect of denosumab treatment, the permanent absence of giant cells associated with severe to mild fibrosis was the most consistent morphological change, but H3F3A positive stromal/tumor cells were observed in all cases. Furthermore, an increased immunopositivity of PDGFRβ in stromal/tumor cells was evident in all recurrent cases during denosumab treatment. Upon tumor recurrence (after the discontinuation of denosumab treatment) the intensity of PDGFRβ immunostaining in stromal/tumor cells was restored/decreased. Our results confirm (for the first time) the activation of PDGFRβ on mononuclear stromal/tumor cells at protein level as an effect of denosumab treatment, which has so far only been demonstrated by phosphoprotein array analysis (protein lysates). The decreased PDGFRβ activity after the discontinuation of denosumab treatmeant and the increased PDGFRβ activity during denosumab treatment underlines the need for denosumab and sunitinib combination therapy.

## Introduction

According to the latest WHO definition, Giant cell tumor of bone (GCTB) is a locally aggressive and rarely metastasizing neoplasm (1). It most frequently occurs in the epiphysis of the long bones of young adults. Histologically, a mononuclear cellular component and osteoclast-like giant cells can be distinguished. The Mononuclear cellular component can be further subdivided into round histiocytic cells (without cytogenetic alterations) and more elongated (oval or spindle-shaped) cells called as neoplastic cells, harboring both numerical chromosomal alterations and H3.3 G34W mutations (2, 3). The gold standard of treatment is surgical: curettage and high-speed burring of the defect with or without the use of adjuvants such as phenol, liquid nitrogen and bone cement (4). Depending on the size and location of the tumor and the type of surgery, the rate of local recurrence is still high (10%–31%). Still, about 95% of cases can be cured with repeated surgery. According to larger series, due to the large destruction of the bone, articular cartilage surface and massive soft tissue component, resection, as primary procedure, might be necessary in about 13%–20% (5, 6). This means however, to sacrifice the adjacent joint and to replace the defect by endoprosthesis in young patients. Furthermore, the rare axial location, the destruction of the sacrum and vertebral body can pose an unsalvageable problem with limited surgical treatment options. In such cases, to avoid mutilating surgery with substantial morbidity, denosumab treatment was launched by Thomas et al (2010) which appeared to be very promising (7). However, several studies have reported that GCTB can recur during/after treatment with denosumab (8-11) which may pose additional challenges. Based on activated receptor tyrosine kinase signaling pattern of stromal/tumor cells, a combination treatment with denosumab and sunitinib has recently been proposed to inhibit recurrences (12). This prompted us to investigate the PDGFRβ expression of five denosumab treated cases using both primary and recurrent tumors during and after denosumab treatment.

### Patients and Methods

#### Patients

Five patients with histologically proven GCTB were selected between 01. 2007 and 12. 2019 from the file of Semmelweis University. Except one case (malignant transformation of GCTB), the original biopsies, as well as the biopsies of the recurrent tumors during denosumab treatment/or immediately after treatment and biopsies of recurrent tumors after at least 6 months of denosumab treatment were available. In the case of the malignant transformation, the original biopsy and the biopsy from malignant GCTB/osteosarcoma (during denosumab treatment) were examined. Clinical data are summarized in Table 1. All five patients had a “high risk tumor” presented with extensive bone loss, pathologic fracture, or destruction of the cortical/subchondral bone, so conservative surgery (curettage and bone grafting) and joint salvage were not possible at presentation. Resection of the GCTB would have been associated with potentially poor function and significant morbidity. At least two recurrences were observed in all cases except the GCTB with malignant transformation (with only one recurrence). None of them had a history of malignancy or radiotherapy of the affected bone. The denosumab treatment did not affect dental and jaw conditions, and the patients were not previously treated with bisphosphonate.

**TABLE 1:** Clinical data of denosumab treated patients.

Case number	1	2	3	4	5
Gender	Female	Male	Female	Male	Male
Age (at presentation; years)	20	40	41	39	28
Location	os ischii	dist. femur	dist. tibia	dist. radius	prox. femur
Recurrence	3x	4x	3x	2x	1x*
Pathological fracture	No	yes	no	yes	No
Pain/swelling	Yes	yes	yes	yes	Yes
Enneking classification	aggr.	aggr.	aggr.	aggr.	aggr.
Denosumab treatment time (months)	15	12	24	7	6
Type of reoperation	curettage+ resection	curettage+	curettage+	resection+	resection++
Follow up after denosumab treatment (months)	10	13	24	8	6
Present status	NED	stable disease	stable disease	NED	Died

Abbreviations: dist., distalis; prox., proximalis; *, malignant transformation in recurrence, malignant giant cell tumor of bone/osteosarcoma; aggr., agressive; curettage+, curettage + adjuvant treatment; resection+, resection + fibula autograft; resection++, resection + endoprothesis; NED, no evidence of disease.

All patient gave a written informed consent for denosumab treatment and their consent to the scientific use of the results. The protocol was approved by the Research Ethics Board of the Semmelweis University (TUKEB 155/2012). CT and MRI of the affected area were performed every 3 months in all patients. The images taken before and after the denosumab treatment were compared by expert radiologists.

#### Histology and Immunohistochemistry

H&E slides were evaluated for diagnostic purpose in the initial biopsy, and H3F3A immunohistochemistry was used to confirm the diagnosis of GCTB. Changes in the histology caused by denosumab treatment were noted and followed during treatment and after discontinuation of denosumab treatment.

Immunohistochemistry was performed on deparaffinized, rehydrated sections obtained from a representative formalin-fixed, paraffin-embedded block from each case using antibody-specific epitope retrieval techniques with the Bond-Max (Leica Biosystems, Wetzlar, Germany) automated system for the detection of the following antigens: PDGFRβ (Cell Signaling Technology, rabbit monoclonal IgG, 28E1, 1:100), H3F3A (RevMAb Biosciences, rabbit monoclonal IgG, RM263, 1:300). The immunohistochemical results were scored according to staining intensity and distribution in case of PDGFRβ, and in case of H3F3A the percentage of nuclear positivity was taken into account. Recurrences during denosumab treatment or very shortly after denosumab treatment (less than 1 month) were termed as “recurrence during denosumab treatment” and recurrences after more than 6 months of discontinuation of denosumab treatment were called as “recurrence after denosumab treatment” (Table 2).

**TABLE 2:** Immunohistochemistry results of primary and denosumab treated GCTB samples (whole-slide evaluation).

Case number	PDGFRß	H3F3A (% of mononuclear cells)
1 primary	+, diffuse	80
1 recurrence den.	++/+++, diffuse	50
1 recurrence after den	+, focal	60
2 primary	+, focal	90
2 recurrence den.	+++, diffuse	50
2 recurrence after den.	+/++, diffuse	60
3 primary	+, diffuse	70
3 recurrence den.	++, diffuse	40
3 recurrence after den.	+, focal	50
4 primary	+, focal	80
4 recurrence den.	++, diffuse	50
4 recurrence after den.	+, focal	50
5 primary	+, diffuse	70
5 recurrence den.	+++, focal	20

Abbreviations: recurrence den., recurrence during denosumab treatment; recurrence after den., recurrence after denosumab treatment.

## Results

### Clinical Response

Four patients experienced significant pain relief by the end of the third month after starting the denosumab treatment. The swelling of soft tissues decreased and the movement of the adjacent joint improved.

Comparing CT and MR images before and after denosumab treatment, four patients showed stabilisation (no progression) but only two patients showed a reduction in defect size. The thickness of the cortical bone was increased, the pathologic fractures were healed. Inside of the cystic defect new bone formation, mineralization was detected in every case however, residual cysts varying in size and number remained detectable. In two patients, the indication for surgery was residual cysts filled by solid tissue in another one local recurrence 1 year after finishing the treatment occurred wich needed a resection. One patient had a complex deformity following the healing of his pathological fracture of the distal radius, which led to loss of function of the wrist*.* The defects of the former two patients were curetted rinsed by phenol as adjuvant treatment and filled up by morzelised bone. The distal part of the radius was resected in one patient and autologous fibula was used to replace the defect. As a result of follow up, two patients have no evidence of disease, another two have a stable disease with residual cysts, and one patient died (malignant transformation of GCTB). The patient who died was a 28-year-old male with a large lytic lesion in his proximal femur. To avoid the resection of the proximal femur and scarification of the hip joint, he had denosumab treatment for 6 months. His hip pain ceased after the first month. Seven months after the onset of the treatment, his lower extremities became paretic. MRI revealed metastatic tumorous foci in the third, fourth lumbar vertebral bodies and in the first sacral segment. Decompression and stabilization of these segments were performed and tumor tissue was sent for histological examination. This revealed a malignant transformation, malignant GCTB/osteosarcoma. The patient’s general condition improved, he was able to walk again, but his pain in the left hip joint increased again. Radiography revealed a progression of the defect, tumorous bone production and a pathological fracture. A palliative surgery, conventional hip endoprosthesis was implanted and adjuvant chemotherapy according COSS protocol was started. He died with his disease before his chemotherapy could have been finished.

### Histology, Immunohistochemistry

**FIGURE 1:**
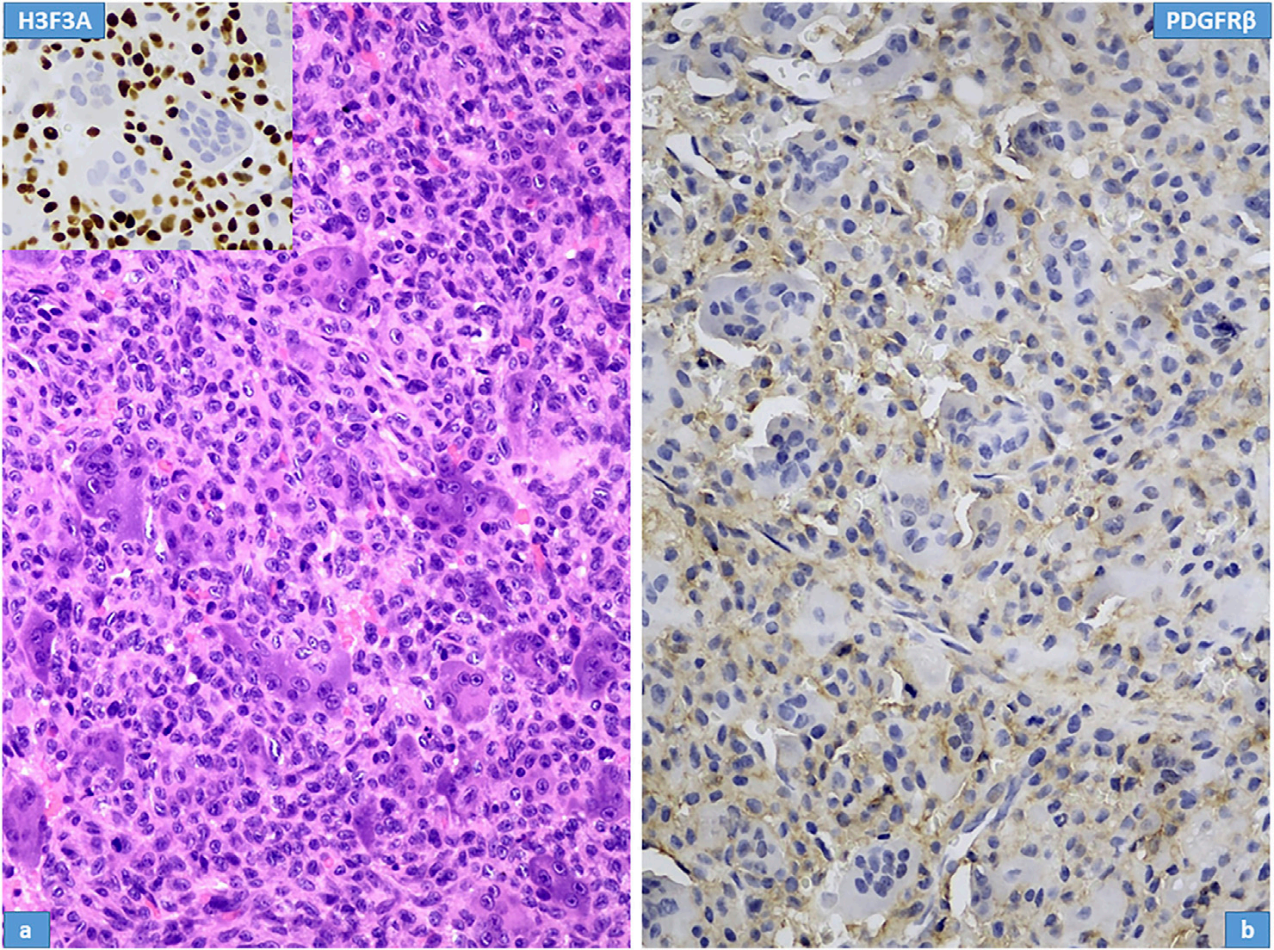
Histopathological features of giant cell tumour of bone; Case Nr: 1. **(A)** The main cell types are stromal cells (histiocytoid mononuclear cells) intermingled with osteoclastic giant cells; the nuclei of both cell types are morphologically similar, with vesicular chromatin and distinct nucleoli. H3F3A immunhistochemical staining (inset) shows intense, extensive nuclear positivity in stromal cells, whereas the nuclei of giant cells are negative. **(B)** PDGFR-beta expression in stromal cells is weak.

**FIGURE 2:**
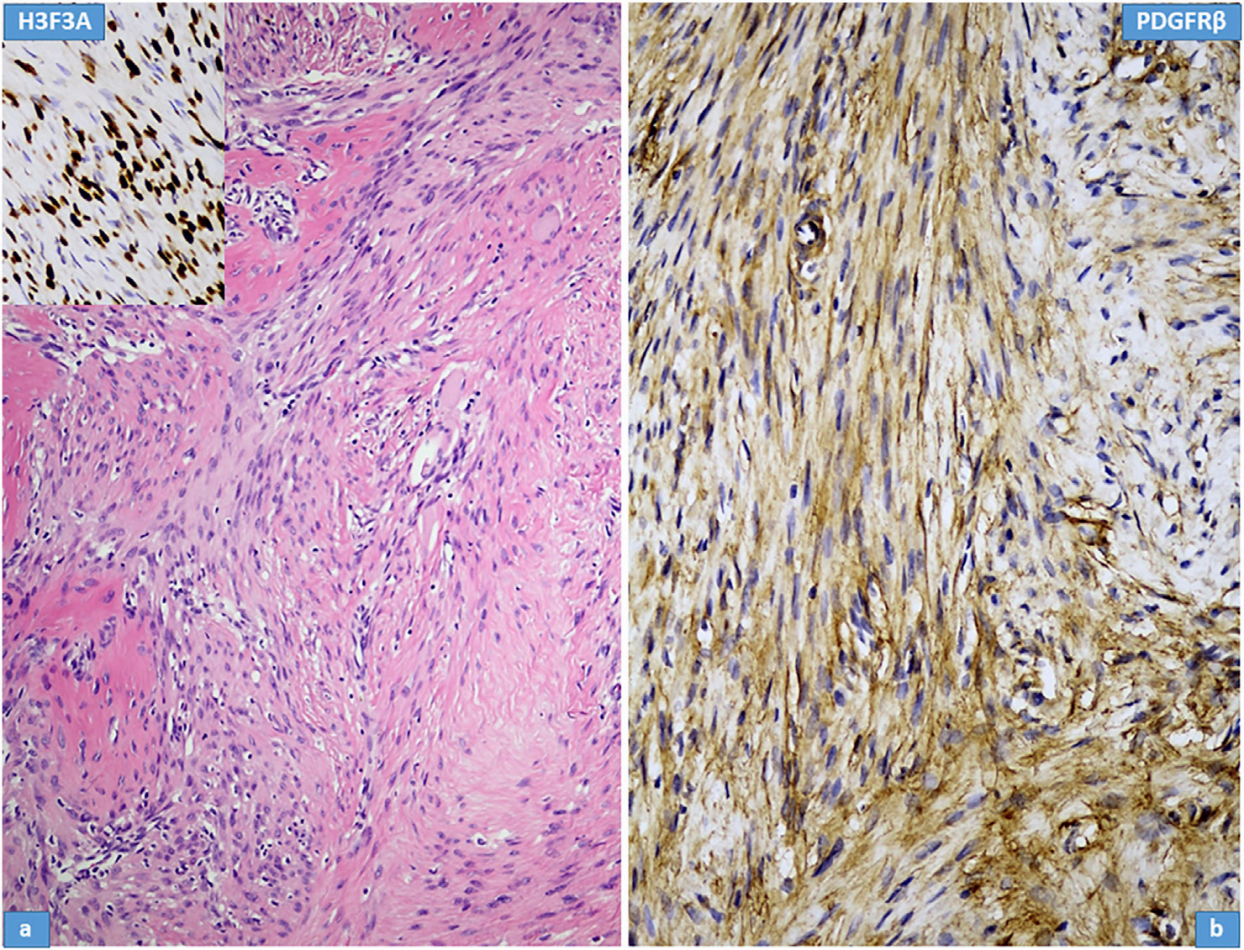
Histological features of recurrent giant cell tumour of bone during denosumab treatment; Case Nr: 1. **(A)** The most important histological features include a reduction in giant cells, a decrease in neoplastic stromal cells, an increase in fibrotic tissue and bone formation. Immunohistochemical staining for H3F3A (inset) is preserved in stromal cells. **(B)** Intense PDGFR-beta expression is observed in stromal cells.

**FIGURE 3:**
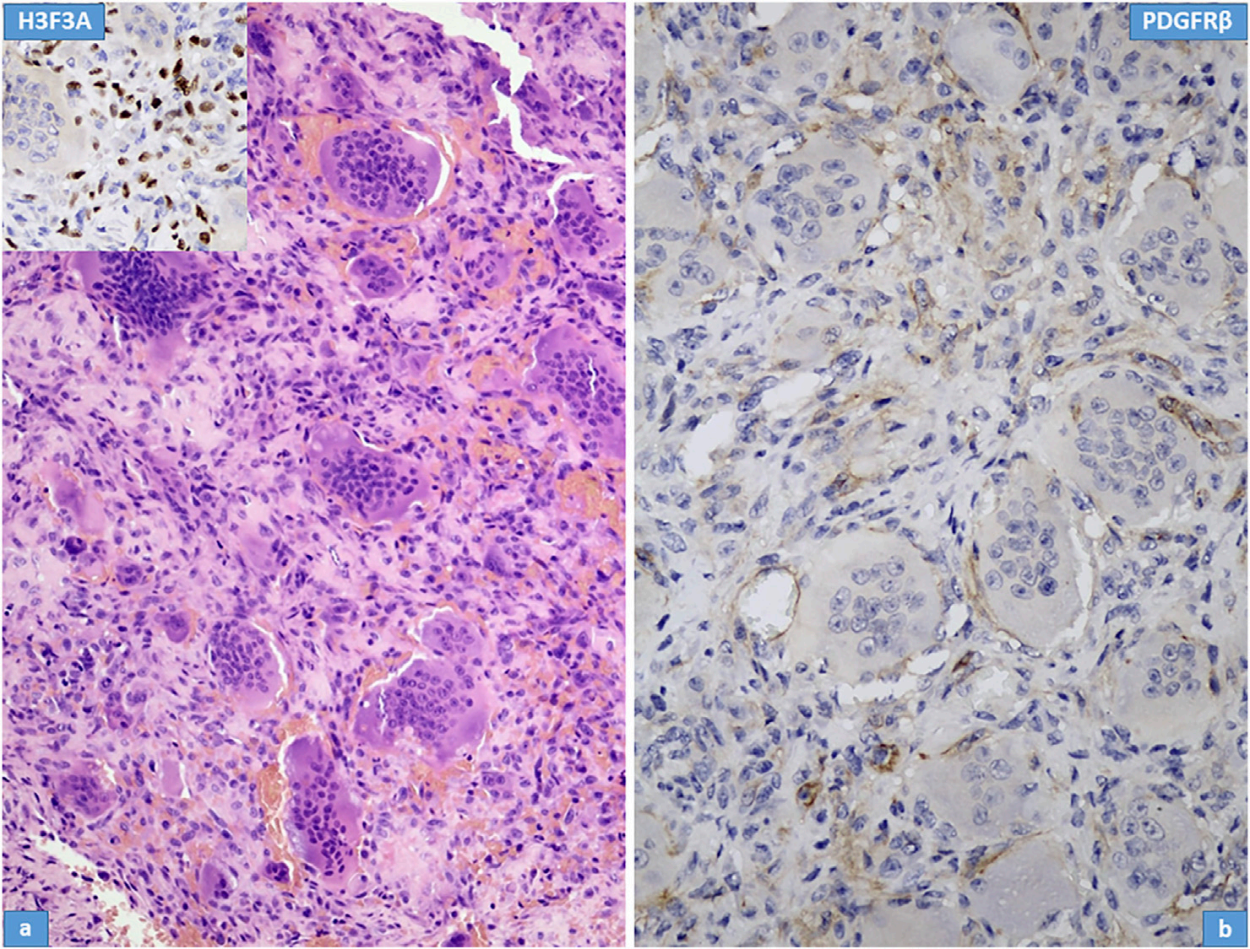
Histological features of recurrent giant cell tumour of bone after denosumab treatment; Case Nr: 1. **(A)** Similar to the primary tumor, giant cells and admixed stromal cells are observed in a mild fibrotic background. Immunohistochemical staining for H3F3A (inset) is preserved in stromal cells. **(B)** Weak PDGFR-beta expression is observed in stromal cells.

**FIGURE 4:**
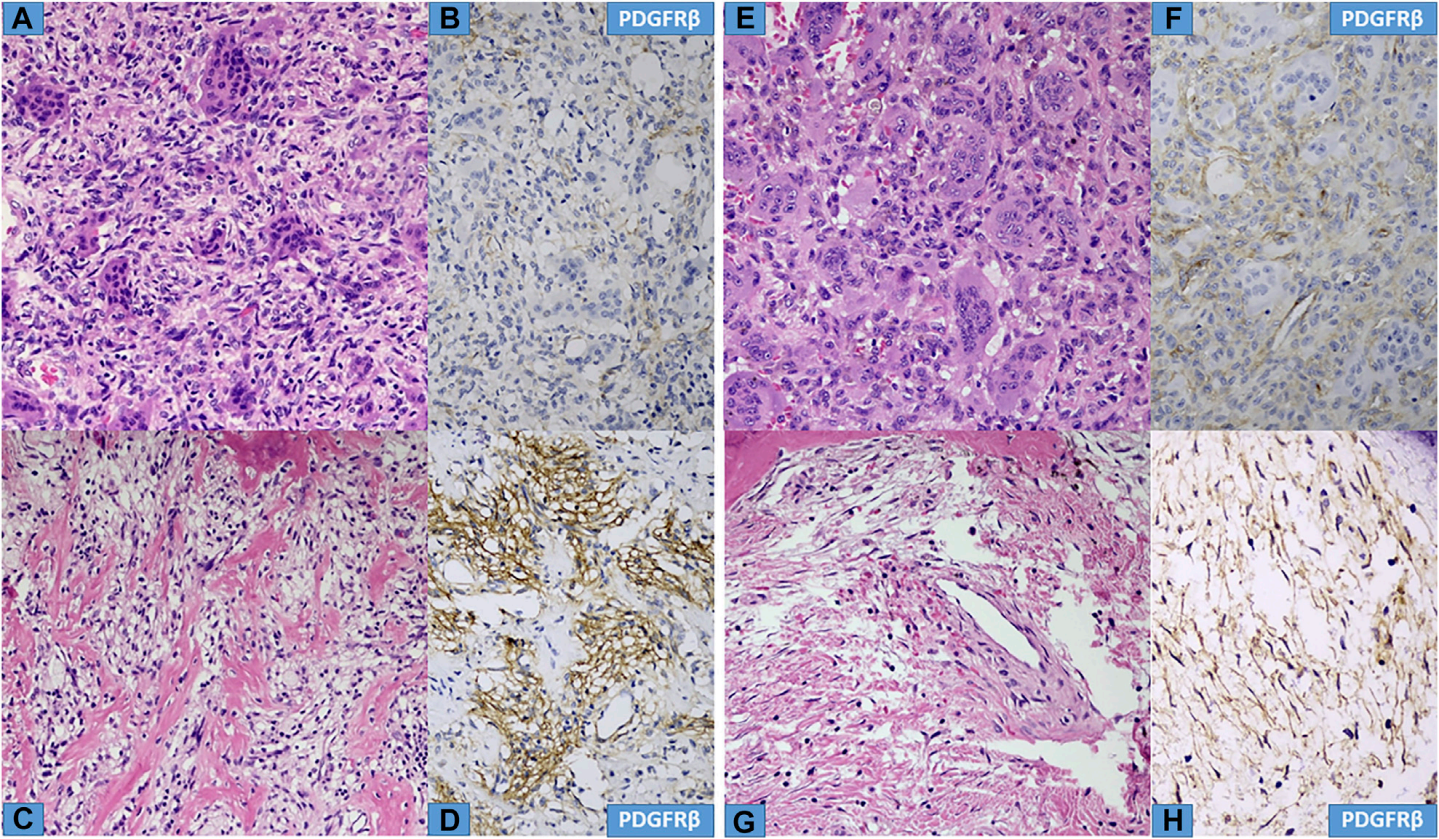
Histological characteristics of Cases Nr: 2 and 3. **(A,E)** Primary tumours, mixed giant cells and stromal cells are observed in a mild fibrotic background. **(B, F)** Weak focal PDGFR beta staining is observed in stromal cells. **(C,G)** During denosumab treatment a decrease in giant cells, surviving neoplastic stromal cells in fibrotic background and bone formation are observed with intense PDGFR-beta expression **(D,H)** in neoplastic stromal cells.

**FIGURE 5:**
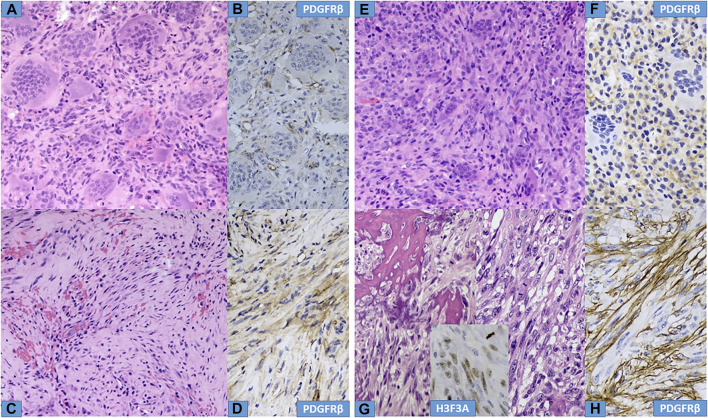
Histological characteristics of Cases Nr: 4 and 5. **(A,E)** Primary tumours, mixed giant cells and stromal cells are observed in a mild fibrotic background. **(B,F)** Weak focal PDGFR-beta staining is observed in stromal cells. **(C,D)** (case Nr: 4) During denosumab treatment a decrease in giant cells, surviving neoplastic stromal cells in fibrotic background are observed with intense PDGFR-beta expression. **(G,H)**. (case Nr: 5) Recurrent tumor during Denosumab treatment showed significant cellular atypia, with increased mitotic figures (malignant GCTB), and intense PDGFR-beta expression in stromal cells. Inset shows focal H3F3A positivity in malignant stromal cells.

Microscopically, the primary biopsy/curettage (in all case) displayed a characteristic admixture of rounded mononuclear histiocytic, spindle-shaped mononuclear neoplastic stromal cells and large reactive multinucleated osteoclast like giant cells, resulting in the diagnosis of GCTB (Figures 1A, 4A,E, 5A,E). In all cases the diagnosis was confirmed by H3F3A immunohistochemistry and the percentage of nuclear positivity of the mononuclear cells were counted. The range was between 70%–90% (Table 2) (Figure 1A inset). Recurrent cases during denosumab treatment showed various histologic picture, but the most consistent alteration was the permanent absence of giant cells. Bone formation, ossification, severe to mild fibrosis, bundles of loose fibrous tissue, thin-walled vessels and a proliferation of mononuclear cells with bland, spindle nuclei were observed in different proportion as a typical response to denosumab treatment (13) (Figures 2A, 4C,G, 5C). In case 5, the recurrent tumor showed a malignant transformation with highly cellular, atypical spindle cell proliferation. Unequivocal tumorous osteoid formation and high mitotic rate (8%–10/HPF) was obvious (Figure 5G). H3F3A immunohistochemistry still was positive in all cases, but in a much lower percentage compared to the primary biopsy/curettage (range: 40%–50%), especially in case of malignant transformation (20%) (Table 2) (Figure 2A inset, 5g inset). The histology of recurrent cases after denosumab treatment was more similar to the histology of primary biopsies than to recurrent tumors during denosumab treatment, but more or less fibrosis and bone formation could be observed in each case (Figure 3A). The percentage of H3F3A positivity was in between of the primary and recurrent (during denosumab treatment) cases, namely 50%–60% (Table 2) (Figure 3A inset). Generally, PDGFRβ immunohistochemistry was positive in each cases including primary and recurrent tumors (both during and after denosumab treatment), but with different intensity and distribution. Primary biopsies displayed only weak positivity with either diffuse or focal distribution (Table 2) (Figures 1B, 4B,F, 5B,F). In contrast, recurrent tumors during denosumab treatment showed moderate to strong PDGFRβ positivity in a diffuse way (Figures 2B, 4D,H, 5D), except the case with malignant transformation, which pointed strong but focal positivity (Figure 5H). Recurrent tumors after denosumab treatment displayed a similar intensity of PDGFRβ immunostaining as it could be observed in the primary biopsies which means, the intensity of PDGFRβ immunostaining in stroma/tumor cells was restored/decreased (Table 2) (Figure 3B). We investigated two cases of reccurent GCTB without denosumab treatment, but we found the same weak PDGFRB positivity in both cases; very similar to the five primary cases demonstrated in Figures 1, 4B,F, 5B, F (Supplementary Figure S1).

## Discussion

GCTB is a locally aggressive and rarely metastasizing neoplasm histologically composed of a mononuclear cellular component (round histiocytes and elongated neoplastic cells) and osteoclast-like giant cells (14). It is still difficult to understand the cell biology of GCTB, that is the interaction of the different cell types, but a generally accepted concept has been described by Forsyth et al (15) recently: there is a crosstalk between m/wt nucleosome H3.3, telomeres and osteoclastogenesis. Extensive studies (16-18) revealed the mechanism of interaction between the macrophage and osteoclast-like cell populations, which express nuclear factor kappa B (RANK) and the stromal cells, which produce RANK ligand (RANKL). Another landmark discovery was the H3.3 G34W mutation, which proved to be characteristic of GCTB by Behjati et al (19). This mutation can be demonstrated by H3F3A immunohistochemistry and proved highly specific and sensitive to both GCTB and malignant GCTB (3). Behjati et al thought that it was possible that histone 3.3 mutations directly led to osteoclast recruitment, for example through alteration of expression of essential osteoclast signals such as RANK ligand or colony stimulating factor 1 (19). However, recently L. Cottone et al (20) found that H3.3 G34W had no impact on the expression of RANKL and osteoprotegerin, as previously was proposed by Behjati et al. Instead, the Signal Peptide CUB Domain And EGF Like Domain Containing 3 (SCUBE3), a secreted member of the transforming growth factor beta (TGFβ) family, was the most significantly downregulated gene in H3.3 G34W compared to H3.3 WT cells. In this way, SCUBE3 is a valid candidate for regulating osteoclast recruitment (20).

Due to the high rate of local recurrence (10%–31%) and the frequently mutilating surgical intervention (about 5%–10%, especially in unresectable cases), new treatment approaches are vital. Denosumab, a pure humanized monoclonal antibody against RANK-L, has been has been the most promising treatment choice. It reduces the number of osteoclast-like giant cells and the mitotic activity in its precursor cells, leading to the reduction of the aggressive osteolytic activity and also facilitates optimal bone remodeling, described by Thomas et al (7). Thomas et al found a positive histological response in 100% and positive radiological response (stable disease) in 66% of the patients, whereas bone repair occurred in 29% of the cases. Unfortunately, recent studies reported frequent recurrences following the denosumab treatment (13, 21, 22). Traub et al (22) in all their 20 patients observed recurrences during/following denosumab treatment despite reduction in pain intensity and positive histologic and radiological response. Matcuk et al (21) described rapid recurrence of GCTB after cessation of long-term denosumab therapy. Although fortunately very rare, malignant transformation of denosumab treated GCTB is now well-established, as recently reported by Palmerini et al (23). Last year (2021), Mahdal et al (12) described sunitinib as a new target for precision medicine treatment of GCTB; found effective in the treatment of neoplastic stromal cells with activated PDGFRβ signaling. They used fresh-frozen tumor samples and tumor-derived cell lines and an increase in PDGFRβ phosphorylation was found in fresh frozen tumour samples, but it was not statistically significant. However, using tyrosine kinase inhibitor sunitinib *in vitro*, a direct inhibition of the stromal cell proliferation and the differentiation of stromal cells into fibroblast-like cells in the tumor tissue samples was also observed. Furthermore, they performed an off-label treatment with sunitinib (combined with denosumab) in a GCTB-diagnosed patient with aggressive course and gained complete remission/healing. These findings (12) prompted us to investigate the PDGFRβ expression of five denosumab treated cases using both primary and recurrent tumors during and after denosumab treatment. Although the number of cases is not high, it is still enough and unique, because we had four cases where the primary tumor, recurrent tumor during denosumab treatment and recurrent tumor after denosumab treatment were available to examine the possible change in the intensity and distribution of PDGFRβ immunostaining. The fifth case demonstrated a malignant transformation in the recurrence as a rare event and because the patient died on his disease, we could examine only the primary and recurrent tumors. The main question was the effect of denosumab treatment on mononuclear tumor cells (characterized by H3F3A immunopositivity), concerning PDGFRβ expression. It was obvious that denosumab treatment facilitated the increase of PDGFRβ expression, both in intensity and distribution of each cases (Table 2) (Figures 1, 2, 4, 5). The fact, that the intensity of PDGFRβ immunostaining in tumor cells was decreased in recurrences after denosumab treatment (Table 2; Figure 3) confirms the causing role of denosumab regarding PDGFRβ overexpression however, the precise mechanism is not known. There is a small debate in the literature regarding the diagnostic role of H3F3A, but we agree (and experienced) with others that practically all GCTBs are H3F3A positive including malignant GCTBs. In our study, we found variable percentage of H3F3A nuclear positivity among mononuclear cells (20–90%), but the tendency was clear: the percentage was lower in denosumab treated cases (Table 2). Explanation of the former finding can be the relative high number of reactive histiocytes, fibroblasts and bone forming osteoblasts as reactive non-neoplastic cells. The H3F3A positive malignant tumor cells in our malignant GCTB with increased PDGFRβ expression further brace the theory that in rare instances H3.3 G34W mutations combined with PDGFRβ overexpression may cause malignant transformation.

## Conclusion

Summing up our findings, we certified the activation/overexpression of PDGFRβ on mononuclear stromal/tumor cells at protein level (paraffin sections) as an effect of denosumab treatment. Our results also support the need to treat the PDGFRβ activated GCTB cases with denosumab and sunatinib as a combination therapy to prevent further recurrences and to prevent an accidental malignant transformation. The decreased PDGFRβ activity after discontinuation of denosumab treatmeant and the increased PDGFRβ activity during denosumab treatment underlines the need for denosumab and sunitinib combination therapy. However, the low case number is certainly a limitation of the study and further confirmation with higher case number could be necessary.

## Data Availability

The raw data supporting the conclusion of this article will be made available by the authors, without undue reservation.

## Supplementary Material

Supplementary Figure 1
**(A,B)** Immunohistochemical reaction of PDGFRB of two recurrent GCTB cases without denosumab treatment. Note the same weak positivity as in Figures 1, 4B,F, 5B,F.
