# Supplementary figures and images for: The Activation of PDGFRβ on Mononuclear Stromal/Tumor Cells in Giant Cell Tumor of Bone After Denosumab Treatment. An Immunohistochemical Study of Five Cases
Source: Pathol Oncol Res. 2022 Aug 24;28:1610633. doi: 10.3389/pore.2022.1610633 (PMC9448856; doi:10.3389/pore.2022.1610633)

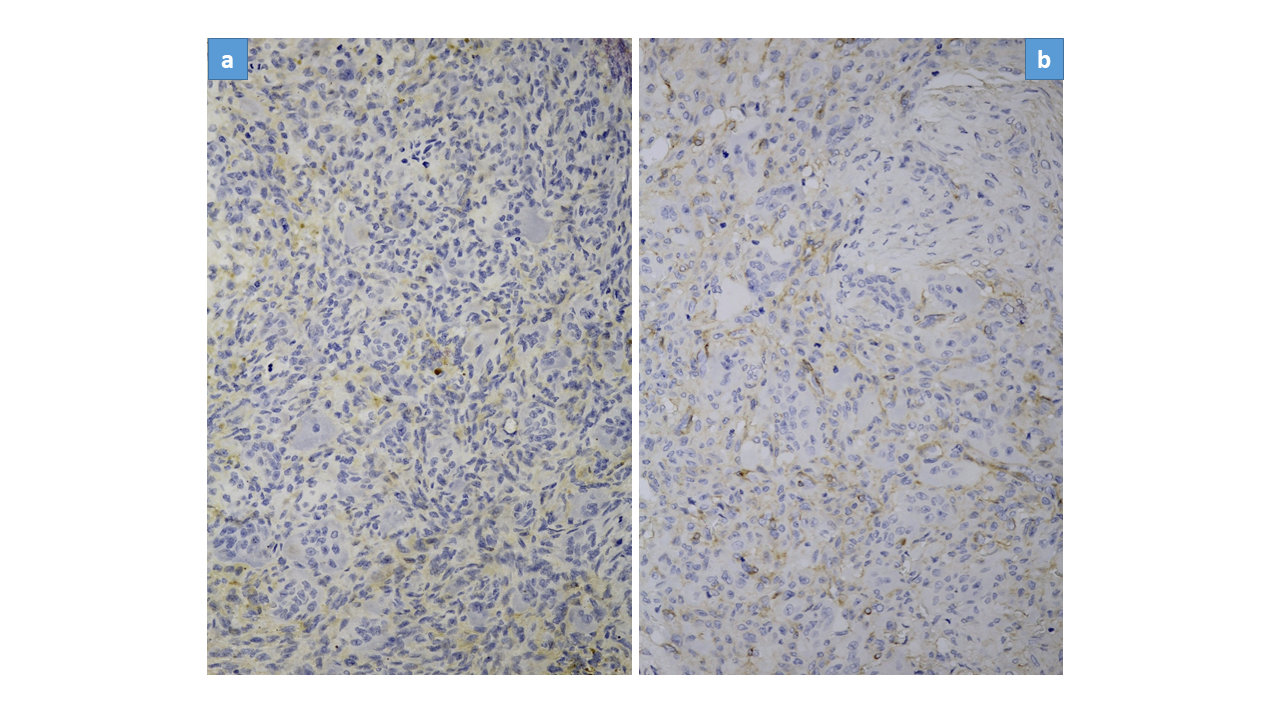

Supplement: Supplementary file 1 [file Image1.TIF]
